# Protective Role for Smooth Muscle Cell Hepcidin in Abdominal Aortic Aneurysm

**DOI:** 10.1161/ATVBAHA.123.319224

**Published:** 2023-04-27

**Authors:** Paul Loick, Goran Hamid Mohammad, Ismail Cassimjee, Anirudh Chandrashekar, Pierfrancesco Lapolla, Alison Carrington, Mayra Vera-Aviles, Ashok Handa, Regent Lee, Samira Lakhal-Littleton

**Affiliations:** Department of Anesthesiology, Intensive Care and Pain Medicine, Universitätsklinikum Münster, Germany (P. Loick).; Department of Physiology, Anatomy and Genetics, University of Oxford, United Kingdom (G.H.M., A. Carrington, M.V.-A., S.L.-L.).; Nuffield Department of Surgical Sciences, University of Oxford, John Radcliffe Hospital, United Kingdom (I.C., A. Chandrashekar, P. Lapolla, A.H., R.L.).

**Keywords:** abdominal aortic aneurysm, ferroportin, hepcidin, neutrophils, smooth muscle cells

## Abstract

Hepcidin is a liver-derived hormone that controls systemic iron homeostasis, by inhibiting the iron exporter ferroportin in the gut and spleen, respective sites of iron absorption and recycling. Hepcidin is also expressed ectopically in the context of cardiovascular disease. However, the precise role of ectopic hepcidin in underlying pathophysiology is unknown. In patients with abdominal aortic aneurysm (AAA), hepcidin is markedly induced in smooth muscle cells (SMCs) of the aneurysm wall and inversely correlated with the expression of LCN2 (lipocalin-2), a protein implicated in AAA pathology. In addition, plasma hepcidin levels were inversely correlated with aneurysm growth, suggesting hepcidin has a potential disease-modifying role.

**Methods:**

To probe the role of SMC-derived hepcidin in the setting of AAA, we applied AngII (Angiotensin-II)-induced AAA model to mice harbouring an inducible, SMC-specific deletion of hepcidin. To determine whether SMC-derived hepcidin acted cell-autonomously, we also used mice harboring an inducible SMC-specific knock-in of hepcidin-resistant ferroportinC326Y. The involvement of LCN2 was established using a LCN2-neutralizing antibody.

**Results:**

Mice with SMC-specific deletion of hepcidin or knock-in of hepcidin-resistant ferroportinC326Y had a heightened AAA phenotype compared with controls. In both models, SMCs exhibited raised ferroportin expression and reduced iron retention, accompanied by failure to suppress LCN2, impaired autophagy in SMCs, and greater aortic neutrophil infiltration. Pretreatment with LCN2-neutralizing antibody restored autophagy, reduced neutrophil infiltration, and prevented the heightened AAA phenotype. Finally, plasma hepcidin levels were consistently lower in mice with SMC-specific deletion of hepcidin than in controls, indicating that SMC-derived hepcidin contributes to the circulating pool in AAA.

**Conclusions:**

Hepcidin elevation in SMCs plays a protective role in the setting of AAA. These findings are the first demonstration of a protective rather than deleterious role for hepcidin in cardiovascular disease. They highlight the need to further explore the prognostic and therapeutic value of hepcidin outside disorders of iron homeostasis.

**Highlights**

- Hepcidin is raised ectopically within smooth muscle cells of the abdominal aorta in the setting of abdominal aortic aneurysm.
- This raised hepcidin plays a protective role by cell-autonomously inhibiting iron export from smooth muscle cells.
- Hepcidin-mediated inhibition of iron export from smooth muscle cells suppresses local lcn2 (lipocalin-2) production.
- Hepcidin-mediated suppression of lcn2 limits neutrophil infiltration into the aortic wall and promotes reparative autophagy in smooth muscle cells.

An abdominal aortic aneurysm (AAA) is an abnormal dilatation of the abdominal aorta, defined as an aortic diameter of >3 cm. Left untreated, an AAA gradually expands and can potentially lead to a catastrophic rupture. Globally, approximately ~200 000 people demise yearly from an AAA.^1^ Patients with AAA are offered an elective repair at 5.5 cm to prevent a future rupture event. In many countries, screening programs exist for at-risk populations.^2^ Traditional risk factors for developing an AAA include cigarette smoking, male gender, age >65, and a family history of AAA. In addition to the risk of AAA rupture, these patients also are at increased risk of mortality from cardiovascular complications.^3^

Hepcidin or HAMP (human antimicrobial peptide) is the master hormone of systemic iron homeostasis. It is primarily produced in the liver and controls systemic iron homeostasis by endocrine inhibition of the iron exporter FPN (ferroportin) in duodenal enterocytes and splenic reticuloendothelial macrophages, respective sites of iron absorption and recycling.^4,5^ HAMP and FPN are also found ectopically in tissues with no recognized role in systemic iron homeostasis, including the heart, the brain, kidney, and pulmonary arterial smooth muscle cells.^6–10^ In the heart and pulmonary arterial smooth muscle cells, work from this laboratory has shown that HAMP acts cell-autonomously to regulate intracellular iron levels, and that such regulation is important for the normal physiological function of the respective tissue.^6,7,10^ HAMP is also induced by inflammation, particularly IL-6 (interleukin-6), accounting for iron deficiency of chronic diseases.^11^ Raised plasma levels of HAMP have been reported in AAA patients.^12^ In addition, there is an association between iron deficiency anemia and increased AAA size and between excess iron deposition within the aneurysm tissue and markers of local oxidative damage.^12,13^ However, the contribution of ectopic HAMP tissue expression to raised plasma HAMP, and its precise role in the pathophysiology of AAA remain unknown.

In this study, we report that both in patients and in an experimental mouse model of AAA, HAMP expression is markedly raised in smooth muscle cells (SMCs) within the aneurysm tissue. In patients, plasma HAMP levels were raised and inversely correlated with aneurysm growth, suggesting a potential disease-modifying role. To probe the disease-significance of raised HAMP in SMCs without the confounding effects of altered systemic iron homeostasis, we generated novel mice harboring an inducible SMC-specific knockout of the *hamp* gene or an inducible SMC-specific knock-in of a HAMP-resistant FPN isoform FPNC326Y. The C326Y mutation conserves the iron export function of FPN but impairs the binding of HAMP, consequently removing the regulation of FPN by HAMP.^7^

## Methods

Reagents are shown in the Major Resources Table in Supplemental Material. Authors will make their data, analytic methods, and study materials available to other researchers upon request.

### Participants, Blood Sampling, and AAA Growth Rate Measurement

All human studies were approved by the local Research Ethics Committee (Reference 13/SC/0250) and conformed to the principles outlined in the Declaration of Helsinki. All participants provided informed written consent. Details regarding the OxAAA (Oxford Abdominal Aortic Aneurysm) Study cohort, recruitment process and relevant ethical approvals have been published.^14,15^ In brief, this single centre prospective study recruited patients in the National Health Service (United Kingdom). Each participant gave written informed consent. Baseline assessments were performed and included demographic and risk factor data, antero-posterior AAA diameter measurements by ultrasound imaging, and fasting venous blood sampling.

Platelet-poor plasma was prepared immediately after blood collection at room temperature using 2-staged centrifugation (first stage: 1300 *g*×12 minutes; second stage: 2500 *g*×15 minutes) as previously described.^15^ These were stored at ≥80 °C for subsequent analysis. Prospective AAA annual growth were calculated based on the antero-posterior diameter measurements in the subsequent yearly AAA monitoring ultrasound scan: (ΔAPD/APD at baseline)/(number-of-days-lapsed/365 days).^16^ To obtain a range of plasma hepcidin values in individuals with no known diagnosis of AAA (healthy controls), hepcidin was measured in a cohort of chronic obstructive pulmonary disease patients who have similar age, BMI, and sex profiles to the AAA group. Patient and healthy control characteristics are shown in Table S1.

### Intraoperative Tissue Biopsy of Omental Artery and Aneurysm Tissue

Intraoperative biopsies of paired tissue samples were performed as previously described.^17^ Tissue samples were prepared immediately in the operating theatre. During AAA surgery, a wedge of abdominal omentum containing a segment of omental artery was identified and biopsied en-bloc. The omental artery segment was cleared of perivascular tissue and snap-frozen. A longitudinal strip of the aneurysm wall along the incision was then excised. The aneurysm tissue was stripped off the perivascular tissue and mural thrombus. The tissue at the maximal dilatation was isolated, divided into smaller segments, and snap-frozen for subsequent analysis. Omental artery and aneurysm wall specimens were also suspended in paraformaldehyde (4%), and later embedded in paraffin moulds. These were sectioned for use in histology and immunohistochemistry experiments. Nonaneurysmal aortas were procured from deceased transplant donors. These were sourced from the Oxford Transplant Biobank (Ethics Ref: 14/SC/1211), which curates tissue samples collected from organ donors. As with aneurysmal and omental tissue, fragments were snap-frozen or fixed and embedded in paraffin for sectioning.

### Mice

All animal procedures were compliant with the UK Home Office Animals (Scientific Procedures) Act 1986 and approved by the University of Oxford Medical Sciences Division Ethical Review Committee.

The conditional hepcidin *hamp* and ferroportin *fpnC326Y* floxed alleles (fl) were generated as described previously.^6,7,10^ Targeting of these conditional alleles to SMCs was achieved by crossing to mice transgenic for tamoxifen-inducible CreER^T2+^ gene under the control of the smooth muscle myh11 (myosin heavy chain polypeptide 11) promoter (SMMHC-CreER^T2+^).^18^ For timed deletion of hamp, and knock-in of fpnC326Y respectively, male hamp^fl/fl^, SMMHC-CreER^T2+^ and fpnC326Y^fl/fl^, SMMHC-CreER^T2+^ animals were treated with an intraperitoneal injection of tamoxifen 5, 3, and 1 days prior to commencing AngII (Angiotensin-II) treatment. Male hamp^fl/fl^ and fpnC326Y^fl/fl^ animals were used as respective littermate controls and also treated with tamoxifen at the same time points. The study was limited to males because the tamoxifen-inducible CreER^T2+^ transgene is on the Y chromosome.^18^

### Hypertension-Induced Mouse Model of AAA-

We used a well-established mouse model of AAA.^19–21^ Recombinant AngII was delivered at a constant dose over several days, using subcutaneously-implanted slow-release Alzet osmotic minipumps (Alzet 1004) as described previously.^19–21^ For implantation of the minipump, animals were anesthetized once for a total of 10 minutes using isoflurane (2% in oxygen) delivered via a nose cone. AngII was infused over 2, 7, or 28 days. The 2-day and 7-day cohorts received an AngII dose of 1 μg/kg/min. A lower dose of 0.33/kg/min was used for the 28-day cohort, to produce a more progressive model of AAA. Animals were culled at the relevant time point by cervical dislocation for collection of plasma and tissues. Animals that died before that time point were subject to postmortem examination to ascertain the cause of death, and in each case, an aneurysm rupture was confirmed by inspection of the aorta in the abdominal cavity. In some animals, LCN2 (lipocalin-2) neutralizing antibody (anti-LCN2) was infused intravenously at 4 mg/kg once, on the same day as the start of AngII infusion.

### Assessment of Aortic Phenotype

Aortic samples from surviving mice at each time point were randomly assigned at harvest either for histology (requiring fixing in formalin and embedding in paraffin) or for gene expression analysis (requiring immediate snap-freezing). For histology, the aortic phenotype was assessed as described previously.^22,23^ Briefly, approximately 1.5 m of the suprarenal aorta was excised, fixed in formalin, then embedded in paraffin. Serial slices were then produced at 1mm intervals, and examined histologically using Van Gieson stain. Slices with the largest lumen size were identified and further sections produced from the corresponding paraffin block for subsequent analysis of the aortic phenotype. Images of Van Gieson-stained aortas were obtained by brightfield microscope and analyzed by ImageJ, to obtain aortic perimeter, from which aortic lumen size and diameter were calculated. To safeguard against any confounding effects of fixation and sectioning artefacts on aortic size data, all sections were produced on the same Leica Jung RM2035 microtome, by the same researcher, who also carried out the staining and Image J quantitation. Elastin integrity was assessed by examining x60 magnification images of Van Gieson-stained aortas, and averaging the number of elastin breaks from a minimum of 10 frames per mouse.

### Quantitative PCR

Once aortic phenotyping studies were complete, additional animal cohorts were run to provide further samples for RNA analysis where necessary. Total RNA extraction and cDNA synthesis were carried out as previously described.^6,7,10^ Gene expression was measured using Applied Biosystems Taqman gene expression assay probes for Hamp, Transferrin receptor (tfr1), Divalent metal transporter (dmt1), lipocalin-2 (lcn2), Beclin1 (beclin1), Microtubule-associated proteins 1A/1B light chain 3B (lc3b) and house-keeping gene β-Actin (Life Technologies, Carlsbad, CA). The computed tomography (CT) value for the gene of interest was first normalized by deducting the CT value for β-Actin to obtain a delta CT value. Delta CT values of test samples were further normalized to the average delta CT values for control samples to obtain delta delta CT values. Relative gene expression levels were then calculated as 2-^delta deltaCT^.

### Histology and Immunohistochemistry

Formalin-fixed paraffin-embedded tissues were sectioned into 5 µm sections and stored at room temperature. Sections were deparaffinized using xylene and then rehydrated in ethanol. For imaging of aortas, slides were stained for elastin using Van Gieson elastin stain kit (HT25A, Sigma-Aldrich) as per manufacturer’s instructions. For Perls iron stain of whole aortas, freshly isolated aortas were submerged in 1% potassium ferricyanide in 0.1 M HCl buffer for 1 hour, washed, pinned onto agarose plates, and then photographed using a standard digital camera. For 3,3’Diaminobenzidine (DAB)-enhanced Perls iron stain, slides were immersed for 1 hour in 1% potassium ferricyanide in 0.1 M HCl buffer and then stained with DAB chromogen substrate. Slides were counterstained with hematoxylin and then visualized using a standard brightfield microscope. For immunostaining, slides were subjected to heat-activated antigen retrieval in citrate buffer (pH 6), then stained with the appropriate antibodies. Antibodies used are detailed in the Major Resources Table in Supplemental Material. Following counter stain with 4′,6-diamidino-2-phenylindole (DAPI), coverslips were visualised using a Fluoview FV1000 confocal microscope (Olympus). All immunostaining experiments included an isotype control, in which slides were stained with the appropriate isotype control (primary antibody) followed by the corresponding secondary antibody. Isotype controls are shown in Figure S1.

### Iron Quantitation

Plasma iron levels were determined using the ABX-Pentra system (Horiba Medical, CA). To quantify iron in tissues, animals were first perfused with saline to remove blood from tissues. Determination of total elemental iron in tissues was carried out by inductively coupled plasma mass spectrometry as described previously.^6,7,10^ Quantitation of ferritin in aortic lysates was determined by Ferritin ELISA (LS-F25188 from LSBio) according to the manufacturer’s instructions.

### Enzyme-Linked Immunoassay

Human HAMP was measured in plasma by ELISA DHP250 (R&D Systems). Mouse HAMP was measured in plasma by ELISA LS-F11620 (Ls-Bio).

### Statistics

Values are shown as mean±SEM. Normality and equal variance were tested using the Shapiro-Wilk test and Levene test respectively. For datasets that passed these tests, pairwise comparisons were performed using 2-sided Student *t* test. Two-factor experimental design data were analyzed by 2-way ANOVA with the Bonferroni Post hoc test. Survival comparisons were performed by Log-Rank (Mantel-Cox) test. For data sets that do not meet normality criteria or with small sample size (≤5), pairwise comparisons were performed by Mann-Whitney Rank-sum test, and multiple comparisons by Kruskal-Wallis 1-way ANOVA test with the Dunn post hoc test. Exact *P* values are shown except when *P<*0.0001. Correlation analysis was drawn using Spearman test. All n values refer to biological replicates. Statistical analyses for mouse data were carried out in GraphPad Prism 9.4.1, and for human data in IBM SPSS V29.

## Results

### HAMP Is Elevated in the Aneurysm Wall and in the Plasma of AAA Patients

**Figure 1.**
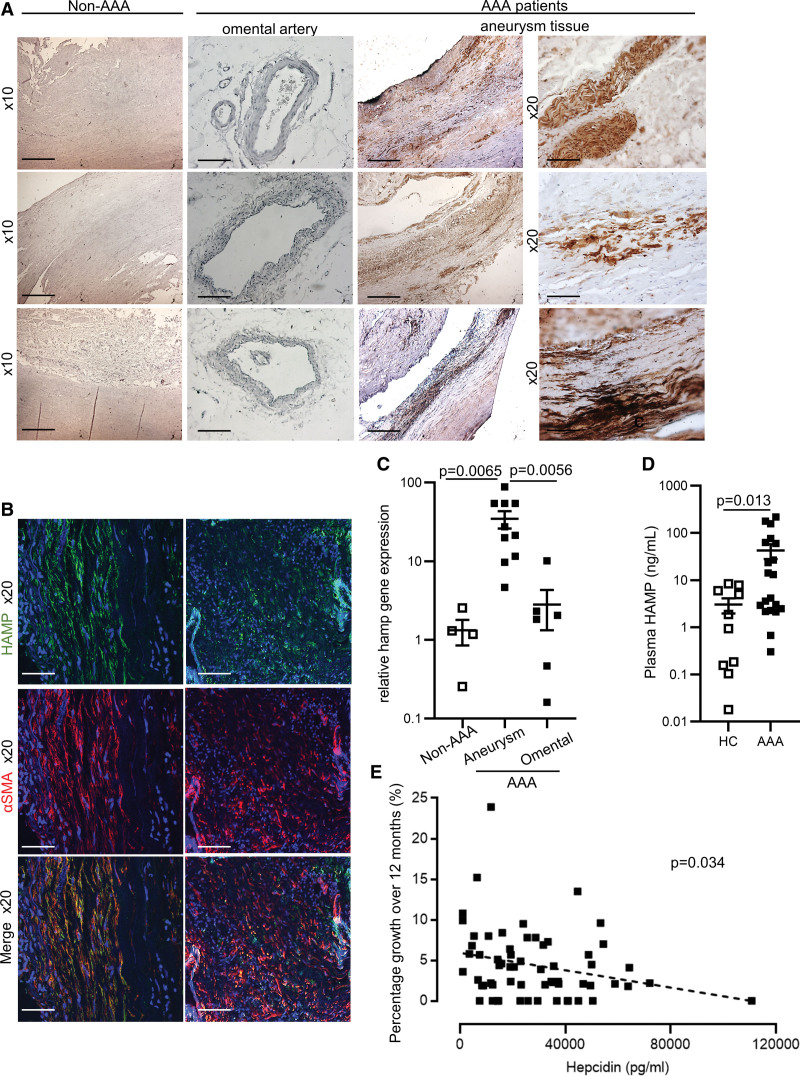
**HAMP (human antimicrobial peptide) is elevated in the aneurysm wall and in the plasma of abdominal aortic aneurysm (AAA) patients. A**, Representative brightfield images of HAMP immunostaining (brown) in abdominal aorta of non-AAA controls (n=4, sex unknown), in omental artery and in aneurysm of AAA patients (7males, 3 females). **B**, Representative immunofluorescence staining for HAMP (green) and the smooth muscle cell marker α-SMA (smooth muscle cell actin alpha; red) in aneurysm of AAA patients. DAPI (blue) was used to identify nuclei. **C**, *Hamp* gene expression in non-AAA controls, in omental artery and in aneurysm of AAA patients (n=4, n=10, n=6, respectively). **D**, Plasma HAMP levels in healthy controls (HCs; n=10) and AAA patients (n=20). **E**, Correlation between plasma HAMP levels at baseline and percentage (%) aneurysm growth over 12 months in AAA patients (n=62). Values are shown at mean±SEM. n refers to biological replicates. *P* values were calculated by Spearman test (**E**), 2-tailed Mann-Whitney *U* test (**D**), Kruskal-Wallis 1-way ANOVA with Dunn post hoc test (**C**). Scale bar=100 μm for original magnification 10×. Scale bar=50 μm for original magnification 20×.

We examined the expression of HAMP in aneurysm tissue excised from AAA patients. We found that HAMP protein was markedly abundant in the aneurysm tissue, in contrast to control vascular tissue (omental artery) from AAA patients and to abdominal aortic tissue from non-AAA controls (Figure 1A). HAMP protein colocalized strongly with SMC marker; SMA-α (smooth muscle cell actin alpha; Figure 1B). There was also some colocalization with the macrophage marker CD68 and the peripheral neutrophil marker Ly6g+Ly6c (Figure S2A). Relative differences in HAMP protein abundance were replicated at the mRNA level, confirming that HAMP protein present in the aneurysm tissue is the product of local gene expression rather than of endocrine source (Figure 1C). When we examined plasma HAMP levels, we found that these were significantly higher in AAA patients than in healthy controls (Figure 1D). Furthermore, higher plasma HAMP concentration at baseline was independently associated with lower aneurysm growth over the subsequent 12 months (Figure 1E). There was no correlation between hepcidin and other demographic variables in this cohort, and the association between hepcidin and aneurysm growth remained significant even after adjusting for potential confounders (Tables S2 and S3). Together, these data suggest that HAMP expression in SMCs of the aneurysm wall may have a disease-modifying role.

### SMC-Derived HAMP Is Protective in the Setting of AAA

**Figure 2.**
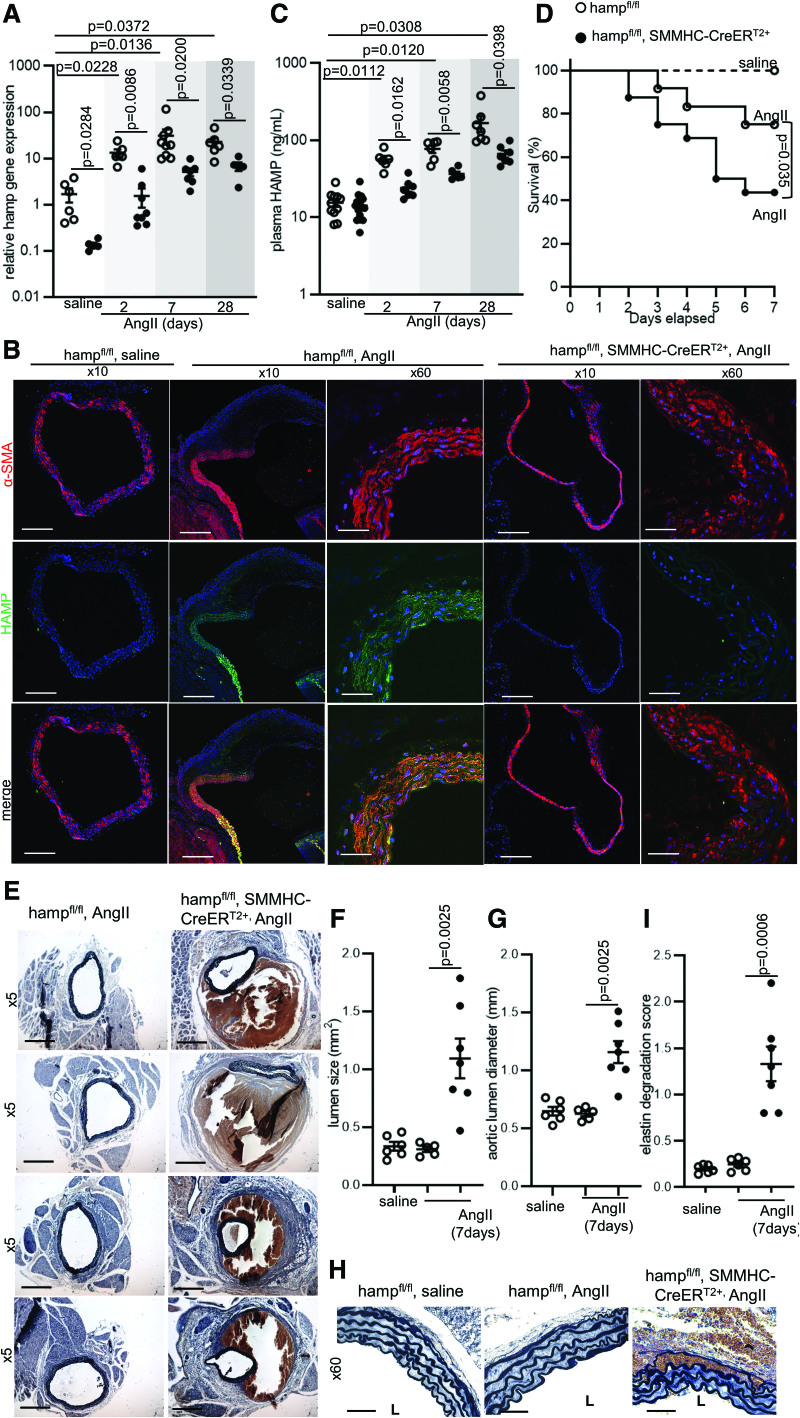
**Smooth muscle cell (SMC)-derived HAMP (human antimicrobial peptide) is protective in the setting of abdominal aortic aneurysm (AAA). A**, Hamp expression in abdominal aorta of hamp^fl/fl^,SMMHC-CreER^T2+^ mice, and hamp^fl/fl^ controls treated with saline or with AngII (Angiotensin-II) for 2, 7, or 28 days, n=5–9 per group. **B**, Representative HAMP (green) immunofluorescence staining in abdominal aorta of at the 7-day time point. α-SMA (smooth muscle cell actin alpha; red) was used to identify smooth muscle cells. DAPI (blue) was used to identify nuclei. n=5–7 per group. **C**, Plasma HAMP levels. n=5–12 per group. **D**, Seven-day survival. n=6–16 per group. **E**, Representative brightfield images of Van Gieson-stained abdominal aortas at the 7-day time point. n=5–7 per group. **F**, Lumen size of abdominal aortas at the 7-day time point. **G**, Aortic lumen diameter in corresponding mice. n=5–7 per group. **H**, Representative brightfield images of Van Gieson elastin stain at the 7-day time point. n=5–7 per group. L=lumen. **I**, Elastin degradation score in corresponding mice from the 7-day time point. n=5–7 per group. Data are shown as mean±SEM. n refers to biological replicates. *P* values were calculated by Log-Rank (Mantel-Cox) test (**D**), 2-way ANOVA test with Bonferroni post hoc test (**A, C**), 2-tailed Mann-Whitney Rank-sum test (**F, G, I**). Scale bar=200 μm for original magnification 5×. Scale bar=100 μm for original magnification 10x. Scale bar=20 μm for original magnification 60×. Isotype controls for immunostaining are shown in Figure S1.

To explore further the potential disease-modifying role of HAMP, we used a well-established experimental mouse model of AAA, in which AngII is delivered subcutaneously via a slow-release pump over several days.^19–21^ Furthermore, to specifically assess the contribution of SMC-derived HAMP, we generated mice harboring a conditional tamoxifen-inducible deletion of the *hamp* gene specifically in SMCs (Hamp^fl/fl^, SMMHC-CreER^T2+^). Efficient deletion of the *hamp* gene in aortas was confirmed at the genomic level (Figure S3A) and these animals did not display any differences in iron parameters or mean arterial blood pressure at baseline (Figure S3B through S3E). In saline-treated animals, *hamp* gene expression in the abdominal aorta was 10-fold lower in Hamp^fl/fl^, SMMHC-CreER^T2+^ mice than in Hamp^fl/fl^ controls, further confirming the efficacy of *hamp* gene knockdown (Figure 2A). *hamp* gene expression in the abdominal aortas was markedly raised after 2, 7, and 28 days of AngII infusion, relative to saline treatment, in both sets of mice. However, this expression remained significantly lower in Hamp^fl/fl^, SMMHC-CreER^T2+^ mice than in Hamp^fl/fl^ controls at each respective time point (Figure 2A). Co-staining with the SMC marker smooth muscle cell actin (α-SMA) confirmed that AngII treatment increased HAMP protein expression in SMCs of Hamp^fl/fl^ mice, but not in Hamp^fl/fl^, SMMHC-CreER^T2+^ mice (Figure 2B). In plasma, HAMP protein levels were not different between saline-treated Hamp^fl/fl^, SMMHC-CreER^T2+^ mice, and saline-treated Hamp^fl/fl^ controls (Figure 2C). Plasma HAMP levels were markedly raised after 2, 7, and 28 days of AngII infusion, relative to saline treatment, but remained significantly lower in Hamp^fl/fl^, SMMHC-CreER^T2+^ mice than in Hamp^fl/fl^ controls at each respective time point (Figure 2C). The liver is normally considered the primary source of plasma HAMP.^4^ However, *hamp* gene expression was not raised by AngII in the liver of either sets of mice (Figure S3F), supporting the notion that raised plasma HAMP following AngII treatment reflects its increased release from SMCs of the aortic wall rather than from hepatocytes.

Having observed this marked increased in SMC hepcidin following AngII treatment, we next set out to determine its role in the pathophysiology of AAA. To that effect, we compared the effects of AngII treatment between Hamp^fl/fl^, SMMHC-CreER^T2+^ mice and Hamp ^fl/fl^ controls. After 7 days of AngII infusion, greater mortality with confirmed aneurysm rupture was observed in Hamp^fl/fl^, SMMHC-CreER^T2+^ mice than in Hamp ^fl/fl^ controls (Figure 2D). Among mice that survived to 7 days of AngII treatment, there was a greater incidence of nonfatal dissection and larger abdominal aortic lumen size and diameter in Hamp^fl/fl^, SMMHC-CreER^T2+^ mice than in Hamp^fl/fl^ controls (Figure 2E, 2F, 2G). Closer examination of the aortas of AngII-treated mice demonstrated marked elastin degradation in Hamp^fl/fl^, SMMHC-CreER^T2+^ compared with Hamp^fl/fl^ controls (Figure 2H and 2I). Similar observations were made in a more progressive AAA model, where animals were treated with a lower dose of AngII for 28 days (Figure S3G through S3K). Together, these data demonstrate that raised HAMP in SMCs of the aneurysm tissue has a protective effect in the setting of AAA.

### The Protective Effect of SMC-Derived HAMP Is Mediated by Its Cell-Autonomous Action on FPN

**Figure 3.**
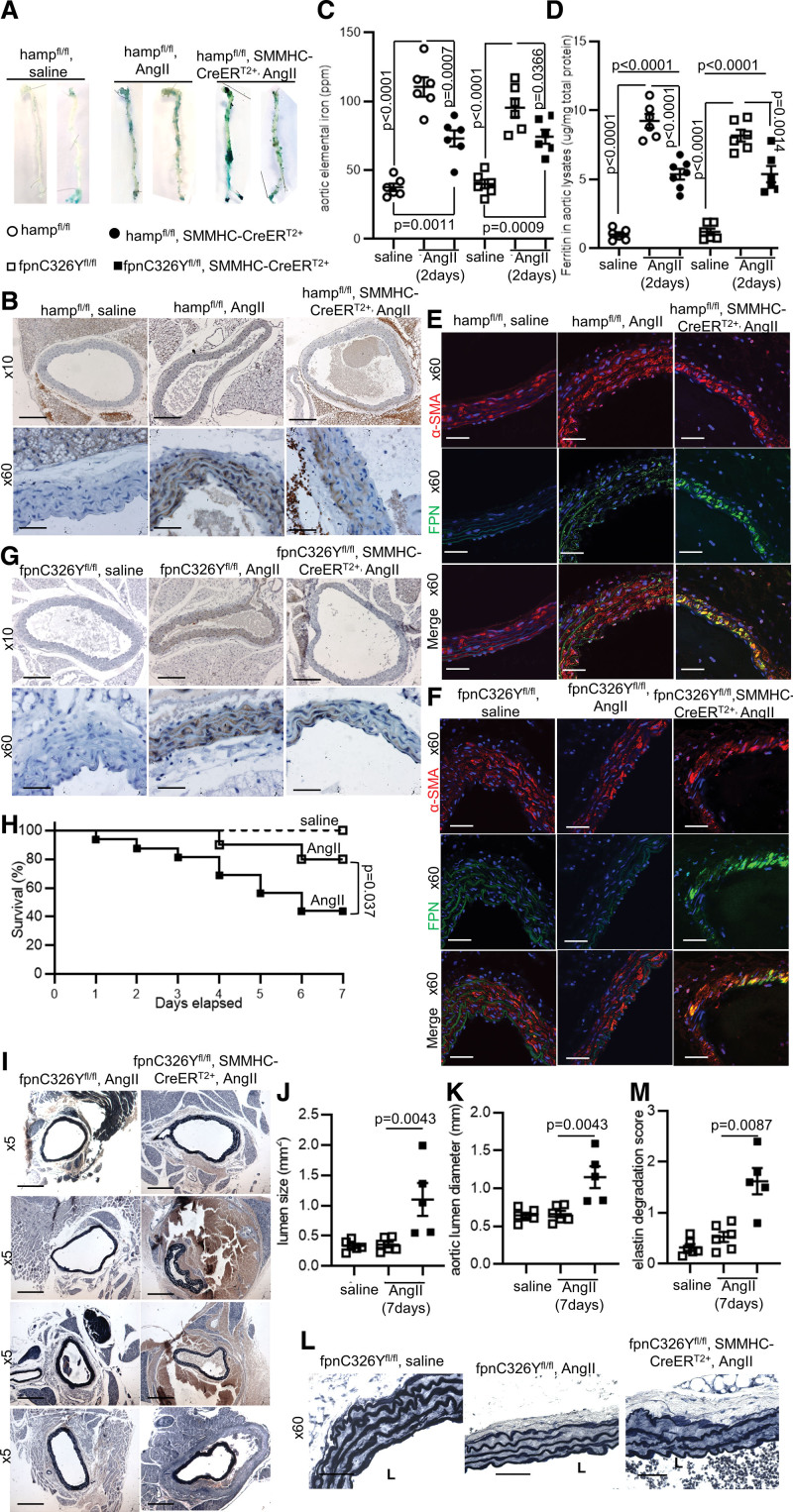
**The protective effect of smooth muscle cell (SMC)-derived HAMP (human antimicrobial peptide) is mediated by its cell-autonomous action on FPN (ferroportin). A**, Representative images of Perls Prussian blue staining for iron in whole aortas from hamp^fl/fl^,SMMHC-CreER^T2+^ mice, and Hamp^fl/fl^ controls after days of AngII treatment. n=6 per group. **B, G**, Representative brightfield images of DAB-enhanced iron stain (brown) in abdominal aortas of hamp^fl/fl^, SMMHC-CreER^T2+^, and fpnC326Y^fl/fl^, SMMHC-CreER^T2+^ mice and respective controls. n=6 per group. **C** and **D**, Quantitation of total iron and of ferritin in abdominal aortic lysates from corresponding mice. n=6 per group. **E** and **F**, Representative immunofluorescence staining for FPN (green) in abdominal aortas. α-SMA (smooth muscle cell actin alpha; red) was used to identify smooth muscle cells. DAPI (blue) was used to identify nuclei. n=6 per group. **H**, Seven-day survival of fpnC326Y^fl/fl^,SMMHC-CreER^T2+^ mice and fpnC326Y^fl/fl^controls. n=6–14 per group. **I**, Representative brightfield images of Van Gieson-stained abdominal aortas at the 7-day time point. n=5–6 per group. **J**, Lumen size of abdominal aortas at the 7-day time point. n=5–6 per group. **K**, Aortic lumen diameter at the 7-day time point. n=5–6 per group. **L**, Representative brightfield images of Van Gieson elastin stain at the 7-day time point. n=5–6 per group. L=lumen. **M**, Elastin degradation score in corresponding mice from the 7-day time point. n=5–6 per group. Data are shown as mean±SEM. n refers to biological replicates. *P* values were calculated by Log-Rank (Mantel-Cox) test (H), 1-way ANOVA with Bonferroni post hoc test (**C** and **D**), 2-tailed Mann-Whitney Rank-sum test (**J, K, M**). Scale bar=200 μm for original magnification 5×. Scale bar=100 μm for original magnification 10×. Scale bar=20 μm for original magnification 60×. Isotype controls for immunostaining are shown in Figure S1.

Having established that SMC-derived HAMP is protective in the setting of AAA, we next set out to determine the mechanism of that protection. First, we examined the levels of iron and of the iron exporter FPN. This is because FPN is the only HAMP target known to date and is inhibited by HAMP at the posttranslational level.^4,5^ To safeguard against any confounding effects secondary to overt AAA pathology, we focused on the earlier time point of 2 days of AngII infusion. At this time point, AngII treatment caused increased iron deposition within the length of aortic wall, evident by Perls Prussian stain of whole aortas (Figure 3A), by DAB-enhanced iron stain of aortic slices (Figure 3B) and by direct quantitation of total iron and ferritin in abdominal aortic lysates (Figure 3C and 3D). However, this iron deposition was quantitively greater in Hamp^fl/fl^ controls than in Hamp^fl/fl^, SMMHC-CreER^T2+^ mice (Figure 3D). Consistent with this, closer examination of the site of iron deposition showed that SMCs of AngII-treated Hamp^fl/fl^ controls showed stronger iron staining than those of Hamp^fl/fl^, SMMHC-CreER^T2+^ mice (Figure 3B). This difference in iron retention within SMCs was consistent with the expression pattern of FPN, which was greater in the SMCs of Hamp^fl/fl^, SMMHC-CreER^T2+^ mice than in Hamp^fl/fl^ controls (Figure 3E). These data indicate that loss of HAMP in SMCs results in increased FPN expression in SMCs, consequently impairing intracellular iron retention. To test this hypothesis, we generated mice harbouring a conditional SMC-specific knock-in of HAMP-resistant ferroportin FPNC326Y (fpnC326Y^fl/fl^, SMMHC-CreER^T2+^). The C326Y mutation conserves the iron export function of FPN but impairs the binding of HAMP, consequently removing the regulation of FPN by HAMP.^7^ As with Hamp^fl/fl^, SMMHC-CreER^T2+^ mice, there was marked upregulation of FPN in SMCs of fpnC326Y^fl/fl^, SMMHC-CreER^T2+^ mice compared with fpnC326Y^fl/fl^, controls (Figure 3F), and this was associated with parallel changes in aortic iron and ferritin levels (Figure 3C, 3D, and 3G). These findings confirm that upregulation of HAMP in SMCs of aortic wall following AngII treatment operates cell-autonomously to inhibit FPN-mediated iron export from SMCs. To determine whether this cell-autonomous action underpins the protective role of SMC-derived HAMP, we examined the aortic phenotype of fpnC326Y^fl/fl^, SMMHC-CreER^T2+^ mice. AngII treatment resulted in greater mortality with confirmed rupture in fpnC326Y^fl/fl^, SMMHC-CreER^T2+^ mice than in fpnC326Y^fl/fl^ controls (Figure 3H). Among the mice that survived to 7 days of AngII treatment, there was a greater incidence of nonfatal dissection and larger abdominal aortic lumen area size and diameter in fpnC326Y^fl/fl^, SMMHC-CreER^T2+^ mice than in fpnC326Y^fl/fl^ controls (Figure 3I through 3K). This was accompanied by more pronounced elastin degradation (Figure 3L and 3M). Thus, loss of HAMP responsiveness in SMCs results in the same heightened AAA phenotype as loss of SMC-derived HAMP. These data confirm that the protective effect of raised SMC-derived HAMP in the setting of AAA is mediated through cell-autonomous inhibition of the iron exporter FPN in SMCs.

### Suppression of LCN2 Contributes to the Protective Effects of SMC-Derived HAMP in the Setting of AAA

**Figure 4.**
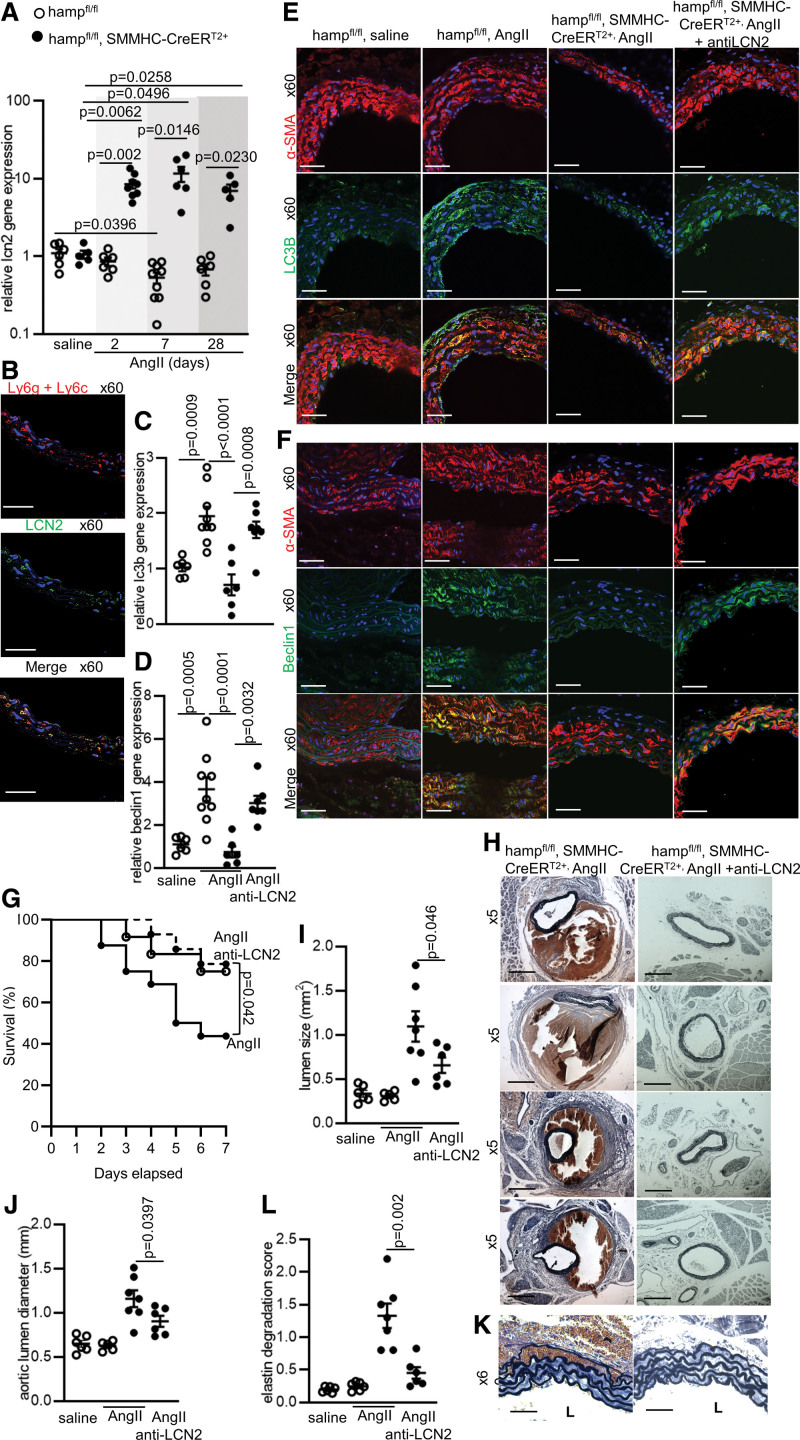
**Suppression of lcn2 (lipocalin-2) contributes to the protective effects of smooth muscle cell (SMC)-derived HAMP (human antimicrobial peptide) in the setting of abdominal aortic aneurysm (AAA). A**, Lipocalin-2 (*lcn2*) gene expression in abdominal aorta of hamp^fl/fl^,SMMHC-CreER^T2+^ mice and hamp^fl/fl^ controls treated with saline or with AngII (angiotensinII) for 2, 7, or 28 days. n=5–9 per group. **B**, Representative immunofluorescence staining for LCN2 (green) in abdominal aorta of hamp^fl/fl^,SMMHC-CreER^T2+^ mice at the 7-day time point. Ly6g+Ly6c (red) was used to identify neutrophils. DAPI (blue) was used to identify nuclei. n=5–7 per group. **C** and **D**, Gene expression of autophagy markers Microtubule-associated proteins 1A/1B light chain 3B (*lc3b*) and *beclin1* in hamp^fl/fl^ controls and hamp^fl/fl^,SMMHC-CreER^T2+^ mice with or without LCN2-neutralising antibody (anti-LCN2) at the 7-day time point. n=6–9 per group. **E** and **F**, Representative immunofluorescence staining for autophagy markers LC3B and Beclin1 (green) in abdominal aorta of corresponding mice. α-SMA (smooth muscle cell actin alpha; red) was used to identify smooth muscle cells. DAPI (blue) was used to identify nuclei. n=5–7 per group. **G**, 7-day survival of hamp^fl/fl^ controls and AngII-treated hamp^fl/fl^,SMMHC-CreER^T2+^ mice with LCN2-neutralizing antibody (closed circles, dashed line) or without LCN2-neutralizing antibody (closed circles, solid line, as per Figure 2D) and of AngII-treated hamp^fl/fl^ controls (open circles, solid line, as per Figure 2D) n=12–16 per group. **H**, Representative brightfield images of Van Gieson-stained abdominal aortas at the 7-day time point. n=5–7 per group. **I**, Lumen size of abdominal aortas at the 7-day time point. n=5–7 per group. **J**, Aortic lumen diameter at the 7-day time point. n=5–7 per group. **K**, Representative brightfield images of Van Gieson elastin stain at the 7-day time point. n=5–7 per group. L=lumen. **L**, Elastin degradation score in corresponding mice from the 7-day time point. n=5–7 per group. To aid comparison, data from saline or AngII-treated hamp^fl/fl^ or hamp^fl/fl^, SMMHC-CreER^T2+^ mice shown previously in Figure 2 are shown again, where appropriate, in Figure 4G through 4L. Data are shown as mean±SEM. n refers to biological replicates. *P* values were calculated by Log-Rank (Mantel-Cox) test (**G**), 2-way ANOVA test with Bonferronis post hoc test (**A**), unpaired 2-tailed *t* test for pairwise comparisons where n>5 (**I, J, L**), 1-way ANOVA with Bonferroni post hoc test (**C** and **D**), Scale bar=200 μm for original magnification 5×. Scale bar=20 μm for original magnification 60×. Isotype controls for immunostaining are shown in Figure S1.

Next, we set out to explore further the mechanisms underlying the protective effect of SMC-derived HAMP by investigating known mediators of AAA pathology. LCN2 is a known mediator of tissue injury in inflammatory disease settings including different forms of vascular.^23–27^ In AAA, LCN2 has been described as having disease-promoting effects.^26,27^ LCN2 has diverse functions, many of which could contribute to AAA. For instance, it has neutrophil-chemotactic properties, and neutrophil infiltration is a well-recognized mechanism of tissue injury in the setting of AAA.^28,29^ In addition, LCN2 has been shown to augment endoplasmic reticulum stress and inhibits reparative autophagy in SMCs and cardiomyocytes respectively,^30–32^ and autophagy is recognized as a reparative mechanism that maintains tissue integrity in the setting of AAA.^33,34^ Our data confirmed that LCN2 is present in the aneurysm tissue of AAA patients, showing some colocalization with the peripheral neutrophil marker Ly6g+Lyc6 (Figure S2B). We also found an inverse relationship between *lcn2* gene expression and *hamp* gene expression in AAA tissues (Figure S2C). Based on these findings, we examined the hypothesis that the protective effect of SMC-derived HAMP involves suppression of LCN2. In mice, we found that *lcn2* gene expression in the abdominal aorta of AngII-treated hamp^fl/fl^ mice was suppressed or unaltered at all time points compared to saline treatment (Figure 4A). However, AngII-treated Hamp^fl/fl^, SMMHC-CreER^T2+^ mice failed to suppress *lcn2* expression, and in fact *lcn2* gene expression was raised rather than suppressed compared to saline-treated animals (Figure 4A). Thus, suppression of local *lcn2* gene expression in AngII-treated mice is dependent on the presence of HAMP in SMCs. When we examined the source of LCN2 in the aneurysm wall, we observed it colocalized with the peripheral neutrophil marker Ly6g+Ly6c (Figure 4B). Next, we examined whether failure to suppress LCN2 was associated with altered levels of autophagy in SMCs or neutrophil infiltration in the aortic wall. We found that AngII treatment induced autophagy in SMCs of hamp^fl/fl^ mice, as evidenced by expression of autophagy markers Microtubule-associated proteins 1A/1B light chain 3B (LC3B) and Beclin1 (Figure 4C through 4F). In comparison, hamp^fl/fl^, SMMHC-CreER^T2+^ mice failed to induce autophagy (Figure 4C through 4F). Furthermore, AngII treatment raised neutrophil infiltration into the aortic wall to a significantly greater extent in hamp^fl/fl^, SMMHC-CreER^T2+^ mice than in hamp^fl/fl^ controls (Figure S4). Autophagy in SMCs was restored (Figure 4C through 4F) and neutrophil infiltration reduced (Figure S4) in hamp^fl/fl^ SMMHC-CreER^T2+^ mice treated with a LCN2-neutralizing antibody (anti-LCN2) at the start of AngII treatment. Anti-LCN2 treatment also reduced the incidence of fatal and nonfatal dissection (Figure 4G and 4H), lowered abdominal aortic lumen size and diameter (Figure 4I and 4J), and prevented elastin degradation (Figure 4K and 4L). To aid comparison, data from saline or AngII-treated hamp^fl/fl^ or hamp^fl/fl^, SMMHC-CreER^T2+^ mice shown previously in Figure 2 are shown again, where appropriate, in Figure 4G through 4L. Together, these data demonstrate that suppression of local LCN2 expression contributes to the protective effect of raised SMC-derived HAMP in the setting of AAA, and that this protective effect likely involves promoting reparative autophagy in SMCs and reducing neutrophil infiltration into the aortic wall

## Discussion

The most important finding of the present study is that the cell-autonomous action of SMC-derived HAMP is protective in the setting of AAA. Indeed, loss of HAMP specifically in SMCs increased the incidence of fatal and nonfatal dissection in experimental mouse model of Ang-II-induced AAA, both in an acute model (high AngII dose over 7 days) and in a progressive model (low AngII dose over 28 days). This phenotype was replicated in a second mouse model, in which SMCs express a HAMP-resistant FPN. Comparing the 2 genetic mouse models used in this study provides the strongest evidence for the cell-autonomous action of SMC-derived HAMP. Indeed, in hamp ^fl/fl^, SMMHC-Cre ER^T2+^ mice, both the autocrine and endocrine actions of SMC-derived HAMP on FPN have been ablated. In fpnC326Y^fl/fl^, SMMHC-Cre ER^T2+^ mice (harboring SMC-specific substitution of wild-type FPN with one that cannot be inhibited by hepcidin), only ablates the autocrine effects of SMC-derived HAMP, while maintaining any endocrine effects (because the *hamp* gene in SMCs is intact in these mice). The finding that these 2 models phenocopy one another in terms of aortic phenotype (ie, heightened AAA phenotype in response to AngII) demonstrates that this heightened AAA phenotype is caused by loss of the autocrine action of SMC-derived HAMP. On the other hand, the finding that these 2 models diverge from one another in terms of the impact of AngII on systemic iron indices (as shown in Figure S5) demonstrates that such systemic effects are dependent on the endocrine function of SMC-derived HAMP.

Another key finding of the present study is that SMC-derived HAMP in the aneurysm tissue contributes to raising plasma HAMP levels, at least in mice. Indeed, plasma HAMP levels were raised in an experimental model of AAA, but only in mice with intact HAMP expression in SMCs, providing the first formal evidence for the contribution of ectopic (non-hepatic) HAMP to plasma HAMP levels. While AAA patients had higher plasma HAMP levels than healthy controls, the tissue source of this plasma HAMP is difficult to ascertain, particularly because these patients likely have confounding co-morbidities that may also contribute to raising HAMP. Our data show that HAMP is raised not only in SMCs but also in immune cells such as macrophages and neutrophils (Figure S2A), which may also contribute to plasma HAMP. Regardless of the source of plasma HAMP in patients, the finding that it was independently inversely correlated with subsequent aneurysm growth supports the notion that it plays a disease-modifying role in the context of AAA.

The mechanisms underlying increased hepcidin expression within the aneurysm tissue are not understood. We investigated several potential mechanisms; including direct effects of AngII, IL-6 and iron. No direct effect of AngII on *hamp* gene expression could be seen in vitro on primary mouse SMCs (Figure S6A). IL-6 did raise *hamp gene* expression in primary mouse SMCs (Figure S6C), consistent with the well-recognized direct transcriptional regulation of *hamp* by IL-6.^35^ Excess iron treatment also raised *hamp gene* expression in primary mouse SMCs (Figure S6C). The latter finding suggests that *hamp* in the abdominal aorta may be induced by iron deposition within SMCs as seen in mice (Figure 3A through 3D), and in AAA tissue from patients (Figure S6B). Another possibility is that hepcidin in SMCs is induced by endoplasmic reticulum (ER) stress resulting from the shear stress of raised blood pressure. This possibility is supported by previous reports that hepcidin is regulated by ER stress.^36–38^

Another open question is the mechanism that underpins iron deposition within SMCs of the abdominal aorta. We found that gene expression of the iron uptake proteins trfr1 and dmt1 were increased as early as day 2 of AngII treatment (Figure S6D and S6E). Increased tfr1 seen in this setting is consistent with previous findings by others.^13^ We also found that AngII treatment raised *fpn* gene expression within the abdominal aorta (Figure S6F). Thus, increased iron uptake via TfR1 and DMT1 into SMCs of the aortic wall SMCs, combined with inhibited local iron export (as a result of HAMP-mediated posttranslational inhibition of FPN) likely also explains why iron deposition was greater in SMCs that produce and respond to HAMP than in those that do not (Figure 3A and 3F).

The mechanisms underlying the protective effect of SMC-derived HAMP appear to depend, at least in part, on the suppression of local LCN2 expression. Neutrophils are considered the primary producers of LCN2, although other cell types also produce it at lower levels.^30,31,39,40^ In line with this, we found that LCN2 appeared to be expressed in infiltrating neutrophils in AngII-treated mice (Figure 4B), and in AAA patients (Figure S2B). LCN2 production by neutrophils is known to be induced in inflammatory conditions, through multiple pathways converging on nuclear factor kappa B (NF-κB), which binds directly to the *lcn2* promoter.^39,40^ Iron is also a potent activator NF-κB signalling.^41,42^ Thus, one possible mechanism through which SMC-derived HAMP might suppress local *lcn2* gene expression in the aneurysm tissue, is by sequestering excess iron within SMCs (through cell-autonomous inhibition of FPN), thereby limiting the iron available to infiltrating neutrophils for the activation of NF-κB activation, and the transcription of *lcn2*.

In AAA, LCN2 has been described as having disease-promoting effects.^26,27^ LCN2 has diverse functions, many of which could contribute to AAA, including its neutrophil-chemotactic properties and its inhibitory effect on reparative autophagy.^28–32^ Consistent with these known functions of LCN2, we saw heightened neutrophil infiltration (Figure S4A and S4B) and impaired autophagy (Figure 4C through 4F) in Ang-II-treated hamp^fl/fl^, SMMHC-CreER^T2+^ mice compared with hamp^fl/fl^ controls; effects that were prevented by neutralization of LCN2. Together, these findings give rise to a model in which, HAMP-mediated suppression of local LCN2 expression dampens neutrophil infiltration into the aneurysm wall, and promotes reparative autophagy in SMCs (graphic abstract).

Our findings on the effects of iron availability on neutrophil function mirror previous findings made by others in macrophages. For instance, it has been shown that ubiquitous loss of hepcidin decreases the activation of macrophages within the atherosclerotic plaque by increasing their FPN levels and decreasing their intracellular iron content.^43^ Another study described the detrimental effect of increased iron uptake by macrophages in the context of AAA.^13^ Together, these studies give rise to the notion that iron availability for immune cells within the vascular bed enhances their activation and inflammatory properties, and promotes cardiovascular tissue injury.^44^ Of note, recent evidence has shown that neutrophils have a particularly high cellular demand for iron.^45^

The finding that AngII altered systemic iron indices (Figure S5) raises the wider question of whether there is an interplay between Angiotensin-II signalling and systemic iron homeostasis. This notion is supported by findings from others,^46–48^ and further studies are warranted to explore this potential interplay and identify its mechanisms.

Plasma HAMP is commonly raised in cardiovascular conditions.^49–51^ Historically, this rise has been considered to be deleterious because it drives anaemia, a well-recognized co-morbidity in cardiovascular conditions.^49–51^ The present study is the first demonstration of a protective rather than deleterious role for raised HAMP in the setting of cardiovascular disease. Evidently, the limitations of experimental mouse models of AAA must be taken into account when considering the translational implications of the present study. Indeed, mouse models that fully recapitulate the human AAA pathology, in terms of manifestation and timeline do not currently exist.^52^ Nonetheless, the study’s findings in AAA patients corroborate the protective role of HAMP in this setting, and highlight the need for larger epidemiological studies into the role of HAMP in the aetiology AAA.

Since the discovery of HAMP at the turn of the century, the consensus has been that it derives primarily from the liver and operates solely as an endocrine regulator of systemic iron homeostasis. In recent years, work from this team has offered new understanding by demonstrating that ectopic HAMP found in other tissues operates in an autocrine fashion to control local iron homeostasis in a manner that is important for the normal physiological function of those tissues.^6,7,10^ The present study adds a new dimension to that understanding, by demonstrating a potential disease-modifying role for ectopic HAMP. In doing so, it highlights not only the multifaceted roles of HAMP in normal and pathophysiology but also the need to further explore its potential as a therapeutic target and as a prognostic marker in conditions other than disorders of iron homeostasis.

## Article Information

### Acknowledgments

We acknowledge support from the Oxford Transplant Biobank (Sandrine Rendel, Jon Milton and Prof Rutger Ploeg) to enable access to nonaneurysm aortic tissue samples collected for the biobank. We thank Peter Santer (Department of Anaesthesia, Critical Care and Pain Medicine, Beth Israel Deaconess Medical Center, Harvard Medical School, Boston, MA) and Peter Robbins (Department of Physiology, Anatomy and Genetics, University of Oxford, United Kingdom) for sharing data on hepcidin levels in healthy controls (COPD cohort). P.L, G.H. Mohammad, A. Carrington, M. Vera-Aviles, and S. Lakhal-Littleton carried out experiments and analyzed data. I.I. Cassimjee, A. Chandrashekar, P.L, A. Handa, and R. Lee prepared and provided patient samples. R. Lee designed patient study and provided patient-related data. S. Lakhal-Littleton designed mouse studies and wrote article. All authors contributed to reviewing the article.

### Sources of Funding

S Lakhal-Littleton received funding from the British Heart Foundation (FS/12/63/29895), Oxford British Heart Foundation Centre of Research Excellence (HSR00030 and HSR00031) and Medical Research Council (MR/V009567/1). R. Lee received funding from the Academy of Medical Sciences Starter Grant (SGL013/1015) and University of Oxford, Medical Sciences Division Medical Research Fund (MRF/HT2016/2191). UK Research Innovation Future Leaders Fellowship (MR/V025775/1), Oxfordshire Health Service Research Committee (Ref: 1321), the Academic of Medical Sciences (SGCL13-1015). The Oxford Abdominal Aortic Aneurysm Study is supported by the following: Oxford University Hospitals NHS Foundation Trust Vascular Surgery Unit, University of Oxford, Medical Sciences Division Medical Research Fund (MRF/HT2016/2191); John Fell Oxford University Press Research Fund (142/075); British Heart Foundation Centre of Research Excellence, Oxford (RE/13/1/30181).

### Disclosures

None.

### Supplemental Material

Tables S1–S3

Figures S1–S6

Major Resources Table

## Supplementary Material


