# Supplementary material for: Protective Role for Smooth Muscle Cell Hepcidin in Abdominal Aortic Aneurysm
Source: Arterioscler Thromb Vasc Biol. 2023 Apr 27;43(5):713–25. doi: 10.1161/ATVBAHA.123.319224 (PMC10125116; doi:10.1161/ATVBAHA.123.319224)
Supplement: Supplementary file 1 [file atv-43-713-s001.pdf]

SUPPLEMENTAL MATERIALS

FULL TITLE- PROTECTIVE ROLE FOR SMOOTH MUSCLE CELL HEPCIDIN IN ABDOMINAL AORTIC ANEURYSM

Loick P† [1], Mohammad G PhD† [2], Cassimjee I MBBCh FCS DPhil [3], Chandrashekar A BE [3], Lapolla P MD [3], Carrington A BA [2], Vera-Aviles M PhD[2], Handa A FRCS [3], Lee R FRCS DPhil *[3], Lakhal-Littleton S DPhil*[2]

Universitätsklinikum Münster, Albert-Schweitzer-Campus 1, Gebäude A1, 48149 Münster, Germany

Department of Physiology, Anatomy and Genetics, University of Oxford. Parks Road, OX1 3PT Oxford, United Kingdom

Nuffield Department of Surgical Sciences, University of Oxford, John Radcliffe Hospital, Headington, OX3 9DU Oxford, United Kingdom

	AAA Participants for growth correlation	Plasma sample analysis		
		AAA Participants	Healthy Controls	P value
**Number (male)**	62 (54)	20 (18)	10 (1)	ns**
**Age at consent, years (SD)**	75 (7)	74 (9)	72 (6)	ns*
**AAA size, mm (IQR)**	48 (43-51)	58 (55-75)	NR	
**BMI median (SD)**	28 (4.5)	27 (5)	26 (6)	ns*
**Blood pressure SBP/DBP, mmHg (SD)**	139/78 (17/11)	140/80 (21/12)	NR	
**Smoking status, n (%)**				
Any history of smoking	54 (87)	18 (90)	10 (100)	ns**
Never smoked	8 (13)	2 (10)	0 (0)	ns**
**History of ischaemic heart disease, n (%)**	39 (63)	5 (25)	NR	
MI/ACS/Stable angina	25 (40)	3 (15)	NR	
Coronary intervention / bypass	14 (23)	2 (10)	NR	
**History of cerebral arterial disease, n (%)**	7 (11)	2 (10)	NR	
**History of hypertension, n (%)**	39 (63)	3 (65)	NR	
**History of hypercholesterolemia, n (%)**	42 (68)	8 (40)	NR	
Total cholesterol mmol/L (IQR)	4 (3.3-4.9)	3.8 (3.3-4.8)	NR	
HDL mmol/L (IQR)	1.1 (1-1.4)	1.2 (0.9-1.4)	NR	
LDL mmol/L (IQR)	2 (1.6-3)	2.0 (1.6-3.2)	NR	
Triglycerides mmol/L (IQR)	1.3 (0.9-1.8)	1.3 (0.9-1.5)	NR	
**History of diabetes mellitus, n (%)**	13 (21)	5 (25)	NR	
HbA1C%, mean	6.00%	5.60%	NR	
Oral anti-hyperglycaemic, n (%)	10 (16)	4 (20)	NR	
Insulin, n	0	1	NR	
**Chronic kidney disease (eGFR<60), n (%)**	15 (24)	5 (25)	NR	
Creatinine µmol/L (IQR)	80 (68-95)	82 (67-94)	NR	
**Chronic respiratory disease, n (%)**	13 (21)	5 (25)	10 (100)	p=0.0002**
**Family history of AAA, n (%)**	16 (26)	7 (35)	NR	
**History of treated neoplasms, n (%)**	10 (16)	3 (15)	NR	
**Regular medication, n (%)**			NR	
Aspirin	40 (64)	9 (45)	NR	
Thienopyridine/Cyclopentyl Triazolopyrimidine	9 (14)	- †	NR	
Oral anticoagulants	7 (11)	2 (10)	NR	
Statin	51 (82)	11 (55)	NR	
b-blocker	24 (39)	4 (20)	NR	
ACE inhibitor / ARB	37 (60)	8 (40)	NR	
**C-reactive protein** (mg/L, IQR)	3.7 (1.1-7.2)	3.8 (1.8-7.7)	NR	

Supplemental table S1- Characteristics of AAA patients used for growth correlation (Figure 1E) and of AAA patients and healthy subjects used for plasma analysis (Figure 1D). Normally distributed data is presented in means and standard distributions (SD). Otherwise data is presented as median with interquartile range (IQR). AAA: abdominal aortic aneurysm; BMI: body mass index; SBP/DBP: systolic/diastolic blood pressure; MI: myocardial infarction; ACS: acute coronary syndrome; HDL/LDL: high/low density lipoprotein; eGRF: estimated glomerular filtration rate; ACE: angiotensin converting enzyme. † Antiplatelet therapy (Thienopyridine/Cyclopentyl Triazolopyrimidine, such as clopidogrel) is routinely discontinued at least five days prior to surgical procedure therefore it is not recorded here. * unpaired two-tailed t test with Welch's correction. ** two sided Fisher's exact test

	Participants for growth correlation	Correlation with Hepcidin (Spearman P value)
**Number (Male gender)**	**62 (54)**	0.47
**Age at consent, years (SD)**	**75 (7)**	0.30
**AAA size, mm (IQR)**	**48 (43-51)**	0.46
**AAA growth (%/year,**	**3.7 (1.9-6.8)**	**0.03**
**Height m (SD)**	**1.74 (0.08)**	0.18
**Weight kg (SD)**	**85 (13)**	0.81
**BMI median (SD)**	**28 (4.5)**	0.43
**Blood pressure SBP, mmHg (SD)**	**139 (17)**	0.65
**Blood pressure DBP, mmHg (SD)**	**78 (11)**	0.37
**Smoking status, n (%)**		
Any history of smoking	**54 (87)**	0.23
Never smoked	**8 (13)**	0.23
**History of ischaemic heart disease, n (%)**	**39 (63)**	
MI/ACS/Stable angina	**25 (40)**	0.99
Coronary intervention / bypass	**17 (27)**	0.88
**History of cerebral arterial disease, n (%)**	**7 (11)**	0.14
**History of hypertension, n (%)**	**39 (63)**	0.97
**History of hypercholesterolemia, n (%)**	**42 (68)**	0.33
Total cholesterol mmol/L (IQR)	**4 (3.3-4.9)**	0.75
HDL mmol/L (IQR)	**1.1 (1-1.4)**	0.31
LDL mmol/L (IQR)	**2 (1.6-3)**	0.95
Triglycerides mmol/L (IQR)	**1.3 (0.9-1.8)**	0.30
**History of diabetes mellitus, n (%)**	**13 (21)**	0.74
HbA1C%, mean	**6.0%**	0.07
Oral anti-hyperglycaemic, n (%)	**10 (16)**	0.87
Insulin, n	**0**	-
**Chronic kidney disease (eGFR<60), n (%)**	**15 (24)**	0.48
Creatinine µmol/L (IQR)	**80 (68-95)**	0.30
**Chronic respiratory disease, n (%)**	**13 (21)**	0.35
**Family history of AAA, n (%)**	**16 (26)**	0.68
**History of treated neoplasms, n (%)**	**10 (16)**	0.94
**Regular medication, n (%)**		
Aspirin	**40 (64)**	0.06
Thienopyridine/Cyclopentyl Triazolopyrimidine	**9 (14)**	0.64
Oral anticoagulants	**7 (11)**	0.41
Statin	**51 (82)**	0.40
β-blocker	**24 (39)**	0.94
ACE inhibitor / ARB	**37 (60)**	0.57
**C-reactive protein** (mg/L, IQR)	**3.7 (1.1-7.2)**	0.32

Supplemental table S2: Correlation between the level of Hepcidin and the other demographic variables in AAA participants used in growth correlation analysis. Correlation analysis was drawn using Spearman's test. Spearman Correlation was assessed due to the non-gaussian distribution of most variables.

Regression

Variables Entered/Removed[a]

Model	Variables Entered	Variables Removed	Method
1	Hepcidin	.	Stepwise (Criteria: Probability-of-F-to-enter <= .050, Probability-of-F-to-remove >= .100).

a. Dependent Variable: % Growth Over 12 Months

Model Summary

Model	R	R Square	Adjusted R Square	Std. Error of the Estimate
1	.256[a]	.065	.050	4.25481

a. Predictors: (Constant), Hepcidin

Coefficients[a]

Model		Unstandardized Coefficients B	Unstandardized Coefficients Std. Error	Standardized Coefficients Beta	t	Sig.
1	(Constant)	5.952	.916		6.497	<.001
	Hepcidin	-5.313E-5	.000	-.256	-2.031	.047

a. Dependent Variable: % Growth Over 12 Months

Excluded Variables[a]

Model		Beta In	t	Sig.	Partial Correlation	Collinearity Statistics Tolerance
1	Age at Consent Date	.124[b]	.984	.329	.128	.998
	BMI	-.014[b]	-.111	.912	-.015	.969
	Male	-.110[b]	-.867	.390	-.113	.990
	Aspirin	.070[b]	.545	.588	.071	.972

a. Dependent Variable: % Growth Over 12 Months
b. Predictors in the Model: (Constant), Hepcidin

Supplemental table 3- Stepwise regression analysis to examine if Gender, Age, BMI and Aspirin had confounding effects on the association between plasma hepcidin and future AAA growth. The analyses was performed in IBM SPSS V29. SPSS output is shown.

Supplemental Figure S1

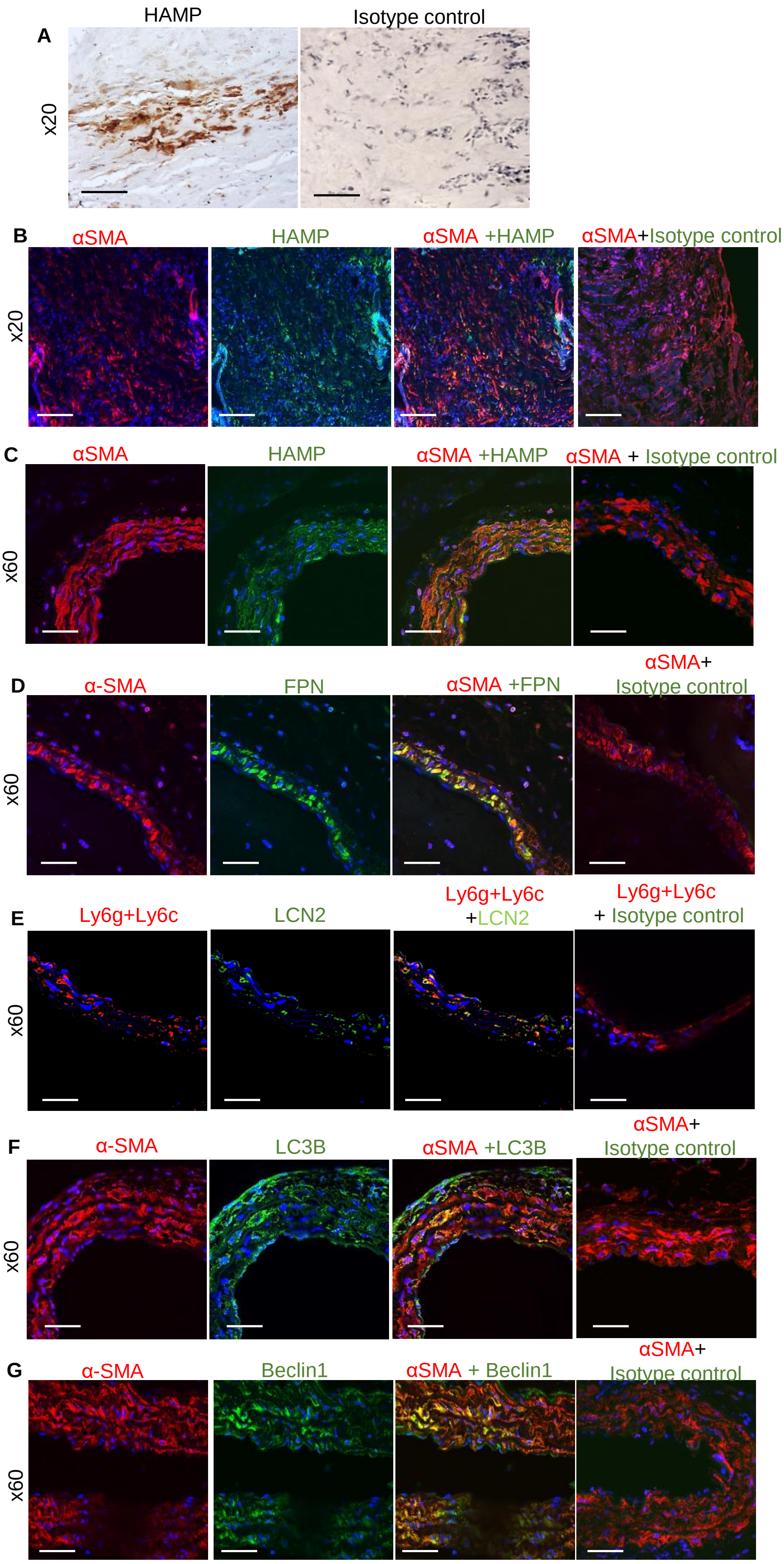

Supplemental Figure S1 – Isotype control immunostainings
A) HAMP immunostaining in human tissues, **B)** HAMP immunofluorescence staining in human tissues, **C)** HAMP immunofluorescence staining in mouse tissues, **D)** FPN immunofluorescence staining in mouse tissues, **E)** LCN2 immunofluorescence staining in mouse tissues, **F)** LC3B immunofluorescence staining in mouse tissues, **G)** Beclin1 immunofluorescence staining in mouse tissues. In each case, slides were stained with the test antibody or with the isotype control from the relevant species, followed with Alexa488-conjugated secondary antibody (green). α-SMA (red) was used to identify smooth muscle cells (B, C, D, F, G). Ly6g+Ly6c (red) was used to identify neutrophils (E). DAPI (blue) was used to identify nuclei. Scale bar=50μm for original magnification 20x. Scale bar=20μm for original magnification 60x.

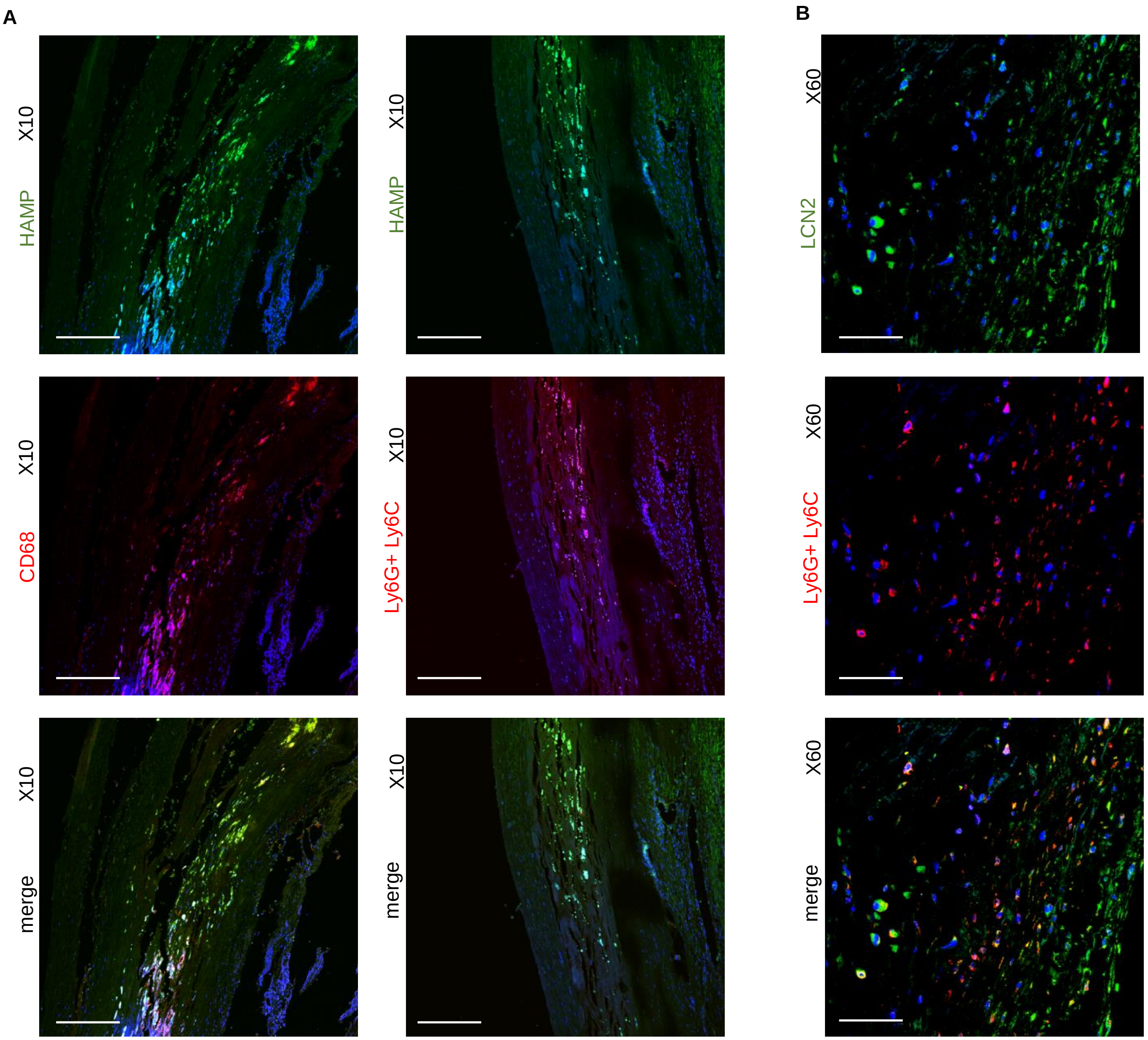

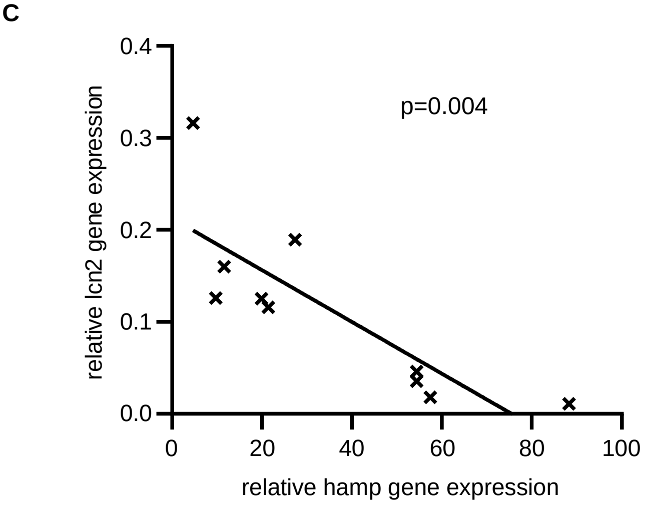

Supplemental figure S2- HAMP and LCN2 in patient AAA tissue. A) Representative immunofluorescence staining for HAMP (green) in aneurysm of abdominal aortic aneurysm (AAA) patients. DAPI (blue) was used to identify nuclei. CD68 staining (red) was used to identify macrophages. Ly6g+Ly6c staining (red) was used to identify neutrophils. Scale bar = 100µm for original magnification 10x. **B)** Representative immunofluorescence staining for LCN2 (green). Ly6g+Ly6c staining (red) was used to identify neutrophils. Scale bar = 20µm for original magnification 60x. **C)** Relationship between *hamp* and *lcn2* gene expression in aneurysm of abdominal aortic aneurysm (AAA) patients. n=10. P values are calculated by Spearman's test. Isotype controls for immunostaining shown in supplemental figure 1.

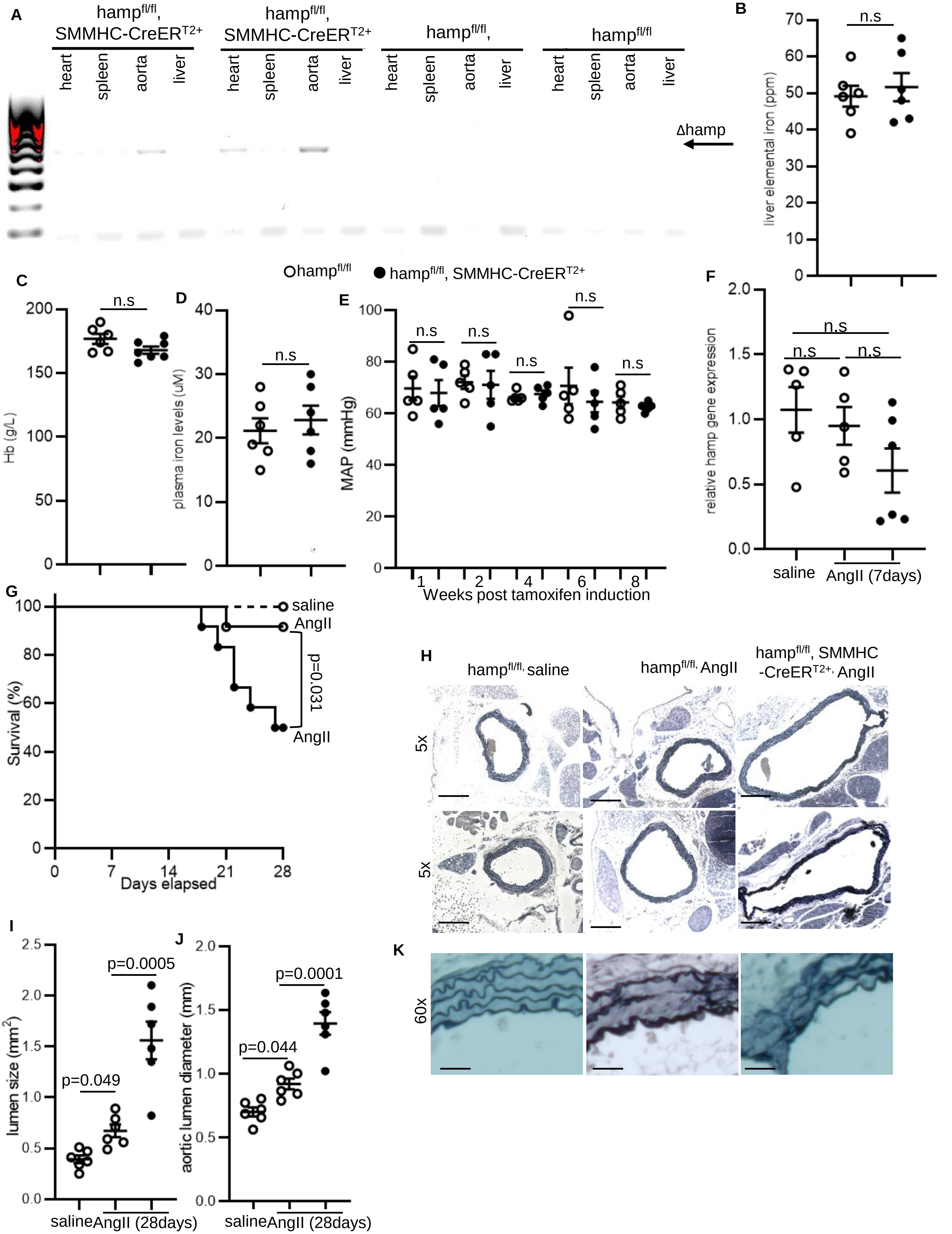
A
hampfl/fl, SMMHC-CreERT2+
hampfl/fl, SMMHC-CreERT2+
hampfl/fl,
hampfl/fl
heart
spleen
aorta
liver
Δhamp
B
liver elemental iron (ppm)
n.s
Ohampfl/fl ● hampfl/fl, SMMHC-CreERT2+
C
Hb (g/L)
n.s
D
plasma iron levels (uM)
n.s
E
MAP (mmHg)
n.s
Weeks post tamoxifen induction
F
relative hamp gene expression
n.s
saline
AngII (7days)
G
Survival (%)
saline
AngII
AngII
p=0.031
Days elapsed
H
hampfl/fl, saline
hampfl/fl, AngII
hampfl/fl, SMMHC -CreERT2+, AngII
5x
I
lumen size (mm2)
p=0.049
p=0.0005
saline
AngII (28days)
J
aortic lumen diameter (mm)
p=0.044
p=0.0001
saline
AngII (28days)
K
60x

Supplemental Figure S3- Characteristics of hamp$^{fl/fl}$,SMMHC-CreER^{T2+} mice . A) Effective gene deletion following tamoxifen treatment was confirmed by detection of deleted HAMP allele (Δhamp) in genomic DNA extracted from abdominal aortas of hamp$^{fl/fl}$,SMMHC-CreER^{T2+} mice compared to hamp$^{fl/fl}$ controls. **B-E)** No differences in baseline levels of liver iron content, haemoglobin, plasma iron levels or mean arterial blood pressure (MAP) between mice of the two genotypes. n=5-7 per group. **F)** No effect of 7-day AngII treatment on hepatic *hamp* gene expression. n=5-6 per group. **G)** 28-day survival of hamp$^{fl/fl}$ controls and hamp$^{fl/fl}$,SMMHC-CreER^{T2+} mice with saline or AngII treatment. n=10-12 per group. **H)** Representative brightfield images of Van Gieson-stained abdominal aortas at the 28-day timepoint. n=5-7 per group. **I)** Lumen size of abdominal aortas at the 28-day timepoint. n=6 per group. **J)** Aortic lumen diameter at the 28-day timepoint. n=6 per group. **K)** Representative brightfield images of Van Gieson elastin stain at the 28-day timepoint. n=6 per group. L=lumen. Data are shown as mean±S.E.M. n refers to biological replicates. n.s= not significant as tested by 2-sided Student t test (B, C, D) and by Kruskal-Wallis one-way ANOVA test with Dunn post hoc test (E). P value calculated by Log-Rank (Mantel-Cox) test (G) and by two-tailed student t test (B, C, D), Mann- Whitney test (E), Kruskal-Wallis one-way ANOVA with Dunn's test (F), one-way ANOVA with Bonferroni correction (I, J). Scale bar=200μm for original magnification 5x. Scale bar=20μm for original magnification 60x.

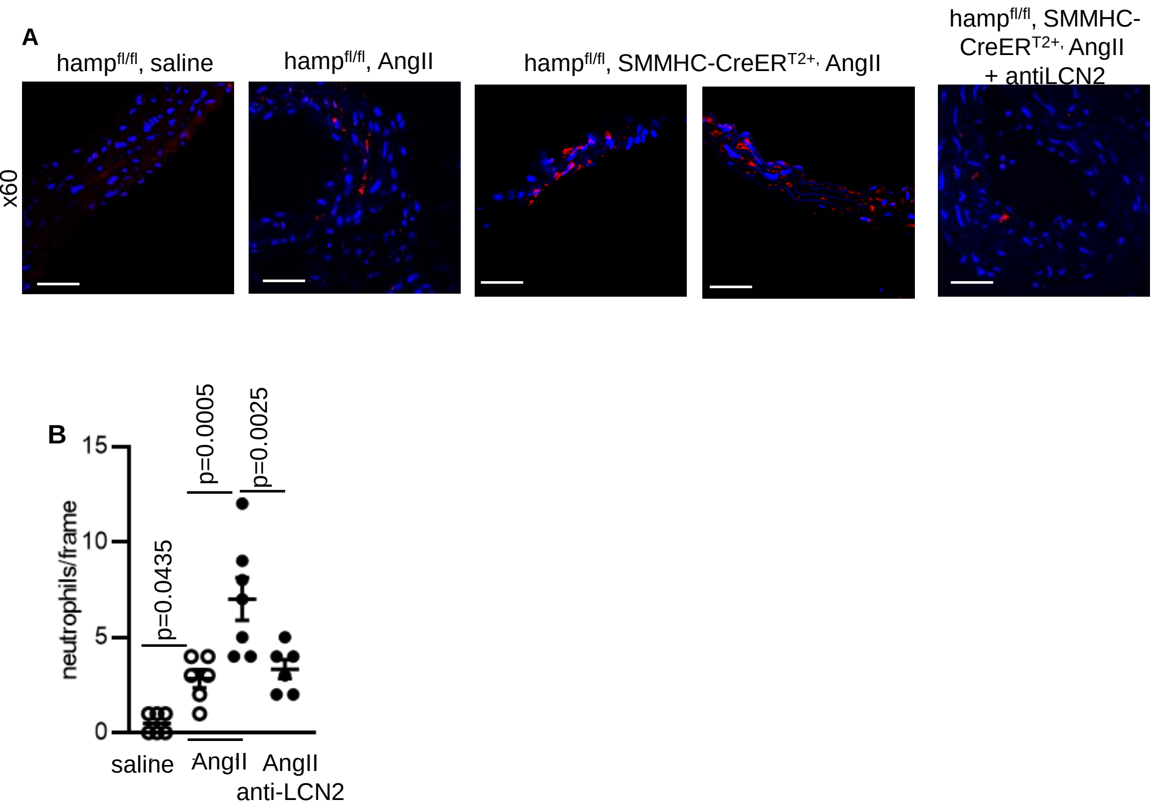

Supplemental Figure S4- Assessment of neutrophil infiltration. A) Representative images of Ly6g+Ly6c immunofluorescence staining (red) in abdominal aortas of $hamp^{fl/fl}$ controls and $hamp^{fl/fl}$,SMMHC-$CreER^{T2+}$ mice treated with saline or AngII with or without LCN2-neutralising antibody (anti-LCN2). n=6-7 per group. DAPI (blue) was used to identify nuclei. **B)** The number of Ly6g+Ly6c positive cells in each frame was counted, and counts averaged across a minimum of 5 frames/mouse. n=6-7 per group. Data are shown as mean±S.E.M. n refers to biological replicates. P values calculated by one-way ANOVA with Bonferroni correction. Scale bar=20μm for original magnification 60x.

Supplemental figure S5

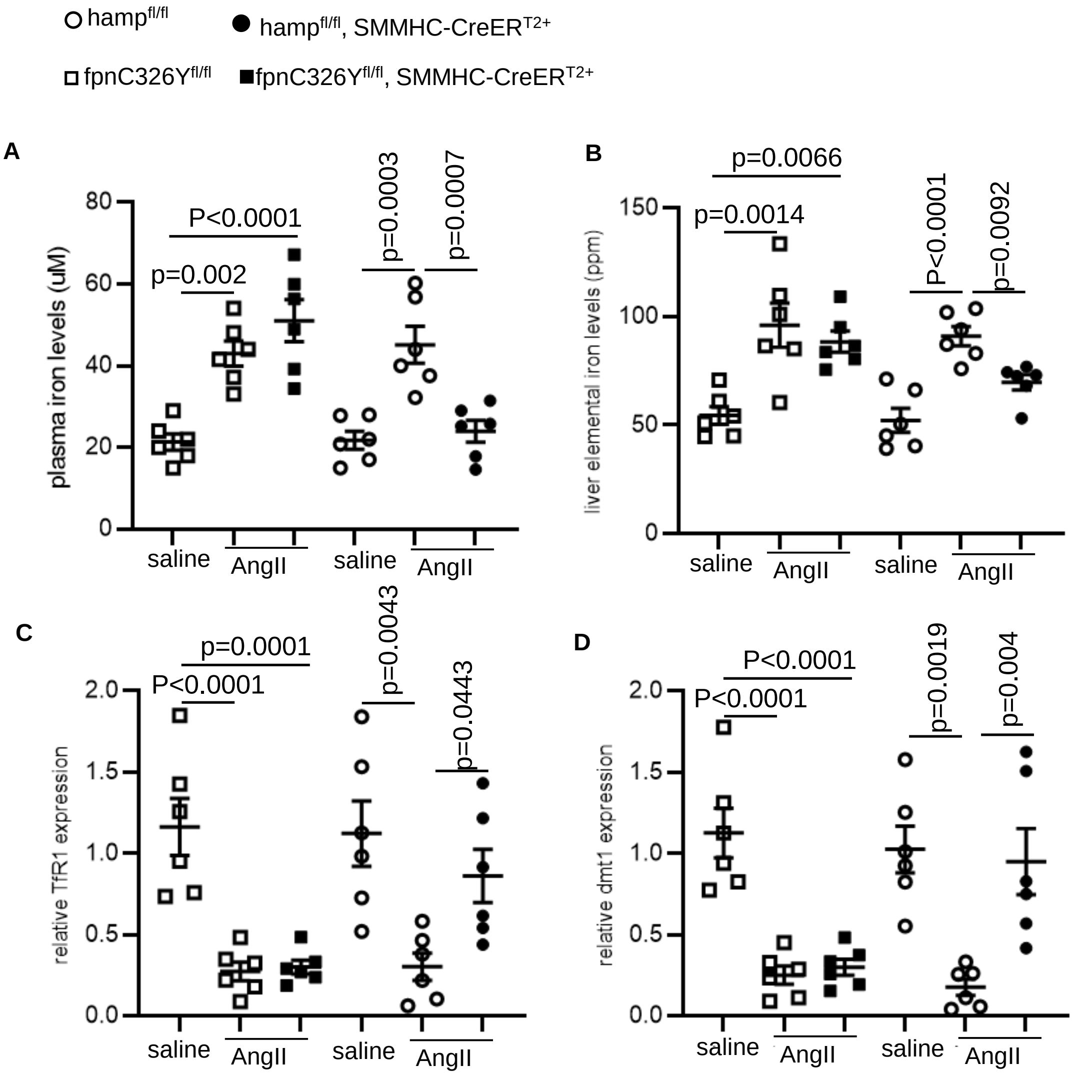

Supplemental Figure S5- Effects of AngII on systemic iron indices. A) Plasma iron levels in fpnC326Y$^{fl/fl}$, SMMHC-CreER^{T2+}and fpnC326Y$^{fl/fl}$ controls, and in and and hamp$^{fl/fl}$, SMMHC-CreER^{T2+} mice and hamp$^{fl/fl}$ controls with saline or AngII treatment (7 days) (n=6 per group). **B)** Elemental iron concentrations in livers of corresponding mice (ppm=parts per million). **C)** Transferrin receptor TfR1 gene expression in livers of corresponding mice. **D)** Divalent metal transport DMT1 gene expression in livers of corresponding mice. Data are shown as mean±S.E.M. n refers to biological replicates. P values calculated by one-way ANOVA with Dunnett's correction.

Supplemental figure S6

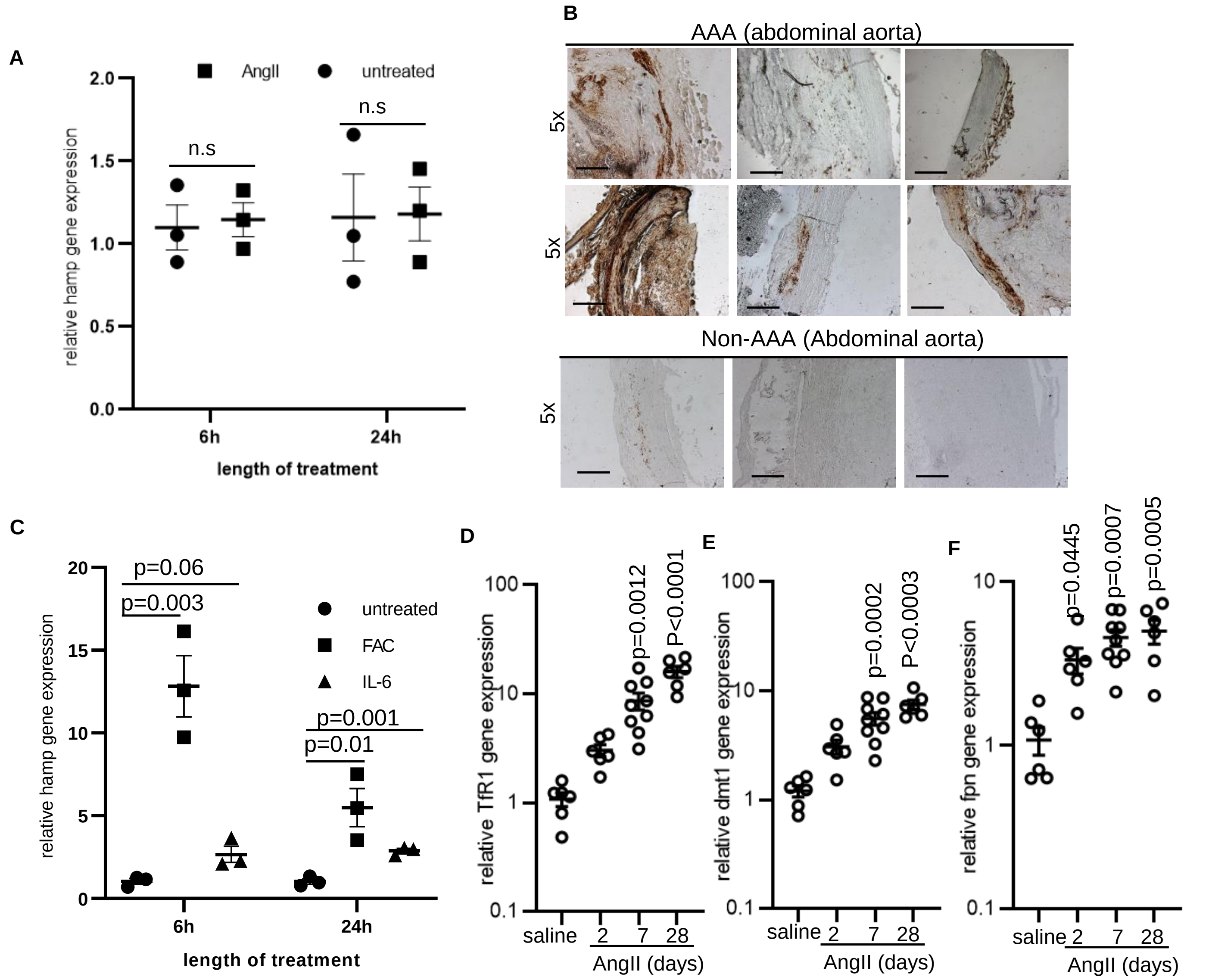

Supplemental Figure S6- Iron and iron regulatory genes in SMCs. A) No direct effect of AngII (100nM) treatment on *hamp* gene expression in mouse primary SMCs. **B)** Representative brightfield images of DAB-enhanced iron stain (brown) in Abdominal aorta from AAA patients and non-AAA controls. **C)** Effect of interleukin-6 (IL-6) at 100ng/mL, and ferric citrate (FAC) at 500uM treatment on hamp gene expression in mouse primary SMCs. **D-F)** Effect of AngII treatment on relative gene expression of Transferrin receptor 1 (TfR1), divalent metal transporter 1 (dmt1) and ferroportin (fpn) in abdominal aortas of mice n=6-9 per group. Data are shown as mean±S.E.M. n refers to biological replicates. n.s= not significant as tested by 2-sided Student t test (A). P values shown in C are calculated by Kruskal-Wallis one-way ANOVA test with Dunn post hoc test (C). P values were calculated by Kruskal-Wallis one-way ANOVA test with Dunnett's correction (D-F). Scale bar=200μm for original magnification 5x

Major Resources Table

Animals (in vivo studies)
Genetically Modified Animals

	Species	Vendor or Source	Background Strain	Other Information	Persistent ID / URL
**Parent - Male**	Mus musculus	N/A (generated in-house)	C57BL/6N		As described in https://doi.org/10.1073/pnas.1422373112
**Parent - Female**	Mus musculus	N/A (generated in-house)	C57BL/6N		As described in https://doi.org/10.1073/pnas.1422373112

Antibodies

Target antigen	Vendor or Source	Catalog #	Working concentration	Persistent ID / URL
HAMP	Abcam	ab30760	2.5ug/mL	https://www.abcam.com/hepcidin-25-antibody-ab30760.html
FPN	Novus Biologicals	NBP1-21502	10ug/mL	https://www.novusbio.com/products/ferroportin-slc40a1-antibody_nbp1-21502h?gclid=EAIaIQobChMI5t-CldS1_QIVx-DtCh1CpwAcEAAYASAAEgL1C_D_BwE&gclsrc=aw.ds#supportresearch
LCN2	Labome	LS-C335204	17.3ug/mL	https://www.lsbio.com/pathplus-antibodies/pathplus-lcn2-antibody-lipocalin-2-antibody-ngal-antibody-if-immunofluorescence-ihc-wb-western-ls-b16528/841323
α-SMA	Sigma	A2547	10ug/mL	Monoclonal Anti-Actin, a-Smooth Muscle clone 1A4, ascites fluid Anti-Alpha Smooth Muscle Actin Antibody (sigmaaldrich.com)
LC3B	Abcam	ab192890	1.9ug/mL	https://www.abcam.com/lc3b-antibody-epr18709-autophagosome-marker-ab192890.html
BECLIN1	Invitrogen	OSA00006W	whole serum, no concentration provided by supplier. Used at 1/1000	https://www.thermofisher.com/antibody/product/Beclin-1-Antibody-Polyclonal/OSA00006W
CD68	Abcam	ab53444	2ug/mL	https://www.abcam.com/cd68-antibody-fa-11-ab53444.html
Ly6g+Ly6c	Abcam	ab25377	0.5ug/mL	https://www.abcam.com/ly6g--ly6c-antibody-rb6-8c5-ab25377.html
Donkey Anti Sheep IgG (Alexa Fluor 488)	Abcam	ab150177	4ug/mL	https://www.abcam.com/donkey-sheep-igg-hl-alexa-fluor-488-ab150177.html

Donkey anti Mouse IgG (Alexa Flour 568)	Abcam	ab175472	4ug/mL	Donkey Anti Mouse (IgG) secondary antibody Alexa Fluor® 568 (ab175472) | Abcam
Donkey Anti rabbit IgG (Alexa Fluor 488)	Abcam	Ab150073	4ug/mL	https://www.abcam.com/donkey-rabbit-igg-hl-alexa-fluor-488-ab150073.html
Donkey Anti rat IgG (Alexa Fluor 568)	Abcam	Ab175475	4ug/mL	https://www.abcam.com/donkey-rat-igg-hl-alexa-fluor-568-preadsorbed-ab175475.html
LCN2 neutralizing antibody	R&D Systems	MAB1857	4mg/Kg	https://www.rndsystems.com/products/mouse-lipocalin-2-ngal-antibody-228418_mab1857

Other

Description	Source / Repository	Persistent ID / URL
AngiotensinII	Merck Life Sciences (A9525)	https://www.sigmaaldrich.com/GB/en/product/sigma/a9525?gclid=EAIaIQobChMIptf9sta1_QIVB-ztCh1DhAxcEAAYASAAEgIG-vD_BwE&gclsrc=aw.ds

Study Design

Study	Groups	Sex	Age	Number (prior to experiment)	Number (after termination)	Littermates (Yes/No)	Other description
Figure 2	hamp$^{fl/fl}$	male	12 weeks	12	12	yes	Received minipumps containing saline
	hamp$^{fl/fl}$	male	12 weeks	40	40	yes	Received minipumps containing AngII dissolved in saline
	hamp$^{fl/fl}$,SMMHC-CreER^{T2}	male	12 weeks	12	12	yes	Received minipumps containing saline
	hamp$^{fl/fl}$,SMMHC-CreER^{T2+}	male	12 weeks	40	40	yes	Received minipumps containing AngII dissolved in saline

Figure 3	FpnC326Y$^{fl/fl}$	male	12 weeks	6	6	yes	Received minipumps containing saline
	FpnC326Y$^{fl/fl}$	male	12 weeks	10	10	yes	Received minipumps containing AngII dissolved in saline
	FpnC326Y$^{fl/fl}$, SMMHC-CreER^{T2+}	male	12 weeks	16	16	yes	Received minipumps containing AngII dissolved in saline
Figure 4	hamp$^{fl/fl}$,SMMHC-CreER^{T2+}	male	12 weeks	12	12	yes	Received a single intravenous infusion of LCN2-neutralising antibody and minipumps containing AngII dissolved in saline

Sample Size:

No prior information on the likely size of the effect of hepcidin gene deletion on the of survival of AngII-treated animals was available. Therefore, a minimum of 10 animals were initially included in the AngII-treatment groups, and 6 in saline-treated groups.

Inclusion Criteria

All males of the desired genotype.

Exclusion Criteria

Females

Randomization

Littermates were assigned to one of two groups according to their genotypes (either cre- or cre+). Mice from each genotype group were then randomly assigned to different treatments.

Blinding

Operator blinded to genotype and treatment group at time of post-mortem examination, serum ELISA assays, histological and immunofluorescent analysis of aortas. Data were unblinded after statistical analysis was completed.
